# Supplementary material for: Socioecological drivers of mutualistic and antagonistic plant-insect interactions and interaction outcomes in suburban landscapes
Source: PLoS One. 2024 Nov 15;19(11):e0312143. doi: 10.1371/journal.pone.0312143 (PMC11567518; doi:10.1371/journal.pone.0312143)
Supplement: S1 Table. L — (DOCX) [file pone.0312143.s001.docx]

Table S1. List of subdivisions (suburban residential developments) included in study.

| **Site ID** | **Subdivision name** | **Latitude** | **Longitude** |
| --- | --- | --- | --- |
| BM1 | Bud Morris Rd^1^ | 36.00664 | 78.61819 |
| BM2 | Hasentree^1^ | 35.98492 | 78.60372 |
| BTS | Bent Tree South | 35.90017 | 78.63192 |
| CB | Wessex | 35.82761 | 78.77958 |
| CCH | Country Club Hill | 35.83361 | 78.65997 |
| CG | Cross Gate | 35.90714 | 78.6365 |
| DP | Dominion Park | 35.91414 | 78.74144 |
| EF | Big Woods (Windy Ridge Rd, Gallup Rd)^2,3^ | 35.79836 | 79.04708 |
| EG | Ethans Glen^2^ | 35.97831 | 78.66881 |
| FOX | Foxcroft | 35.82892 | 78.54411 |
| FR | Falls River^2,4^ | 35.91014 | 78.58942 |
| HP | Heritage Point^5^ | 35.90781 | 78.68036 |
| HS | Hearthstone | 35.70153 | 78.75644 |
| HV | Hidden Valley | 35.87267 | 78.66386 |
| LB | Landings at Lakemoor | 35.68047 | 78.64561 |
| LV | Longview | 35.78311 | 78.60722 |
| LW | Lockwood^6^ | 35.79442 | 78.59867 |
| MC | Mallard Crossing | 35.84653 | 78.49414 |
| MJ | Meadowlands at Jones Dairy | 35.95006 | 78.59878 |
| OT | Olde Town at Rock Quarry | 35.73703 | 78.57783 |
| RV | Lakefall development, Wake Forest | 35.99272 | 78.69858 |
| RW | Riverview | 35.75933 | 78.53806 |
| SC | Stagecoach | 35.63178 | 78.67981 |
| SP | Sheffield Place | 35.91442 | 78.61017 |
| STT | Crockett's Ridge | 35.72086 | 78.89892 |
| TD | Turner Downs | 35.62186 | 78.67675 |
| UM | Village at Pinecrest^6^ | 35.89042 | 78.72092 |
| WR | Wilders Ridge | 35.65639 | 78.64694 |

^1^No data on unmanaged floral resources for this site.

^2^For this site, forest cover was estimated using iTree data [1] rather than land cover data from the National Land Cover Database. We validated our estimate by doing similar estimations for sites where we had land cover data.

^3^For this site, property age was available from tax assessor database. We estimated property age from a Chapel Hill Neighborhoods website. The year homes were built at this site ranged from 1989 to 2002; we selected 1996 as the midpoint of this window.

^4^For this site, only sale price information was available, not the assessed value. We estimated the relationship between property value and sale prices from other sites, using linear regression. Applying this model to homes from FR sold in 2010 yielded a number similar to the average sale price from iMaps in 2022; thus, we used the actual average sale price, rather than our estimate.

^5^For this site, unmanaged floral resources were assessed in only one census rather than two.

^6^Radius surveys (for unmanaged floral resources) were conducted with a single focal plant at this site.

References

1. i-Tree Canopy. Available: http://www.itreetools.org/
