# Supplementary figures and images for: Socioecological drivers of mutualistic and antagonistic plant-insect interactions and interaction outcomes in suburban landscapes
Source: PLoS One. 2024 Nov 15;19(11):e0312143. doi: 10.1371/journal.pone.0312143 (PMC11567518; doi:10.1371/journal.pone.0312143)

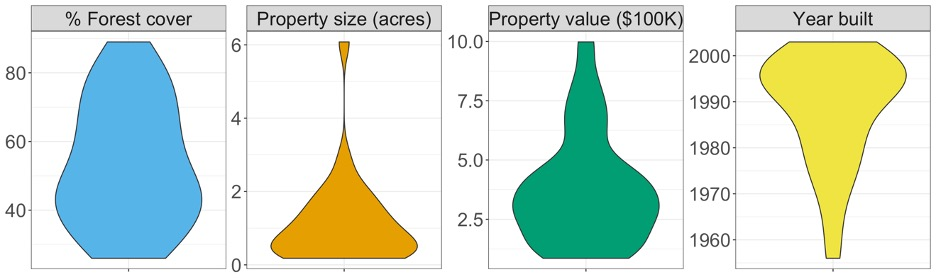

Supplement: S1 Fig — (TIF) [file pone.0312143.s002.tif]

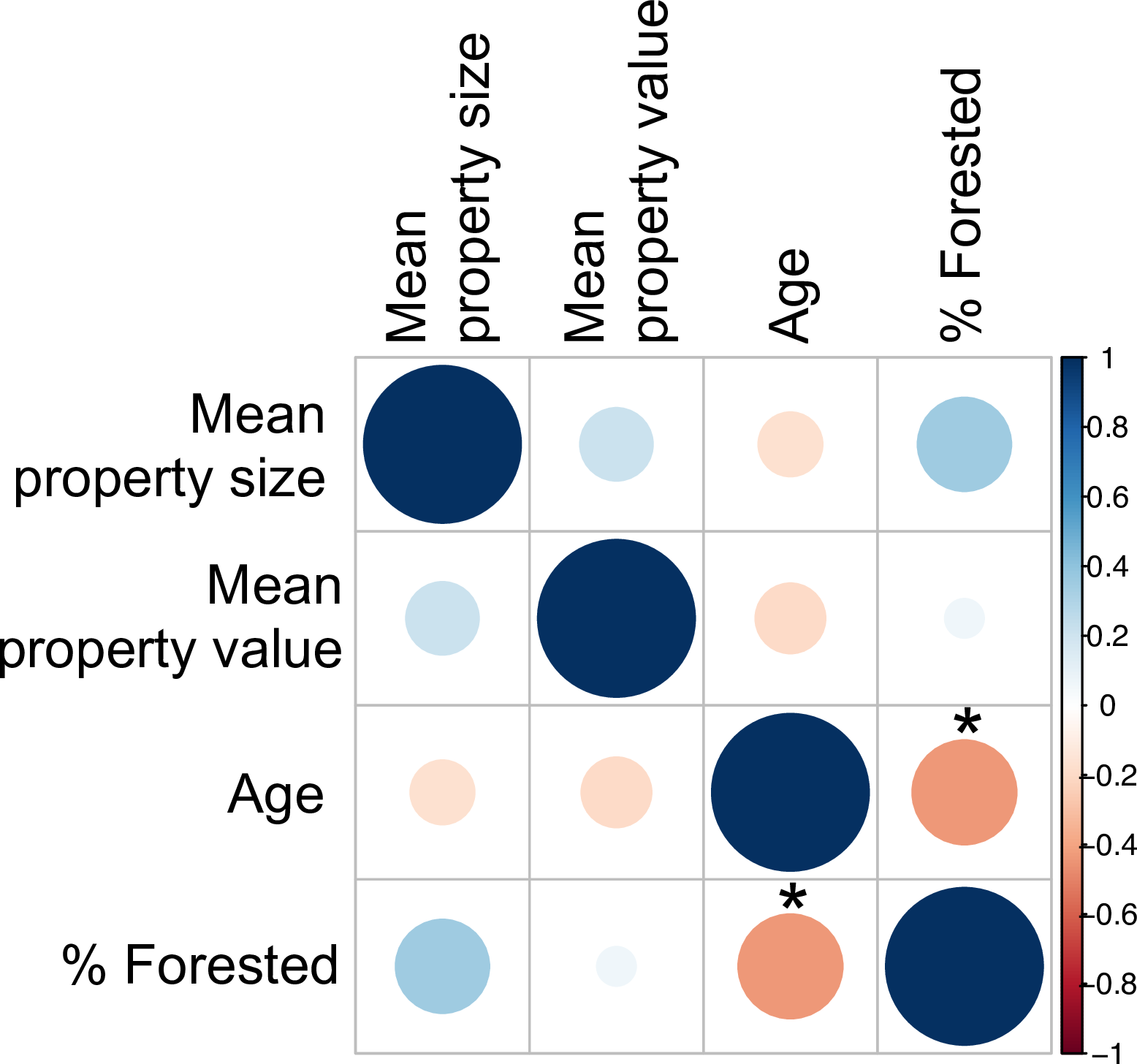

Supplement: S2 Fig — Larger circle and darker color indicate a stronger correlation; positive correlations are shown in blue and negative correlations in red. Significant correlations (p < 0.05) are indicated with an asterisk. (TIF) [file pone.0312143.s003.tif]

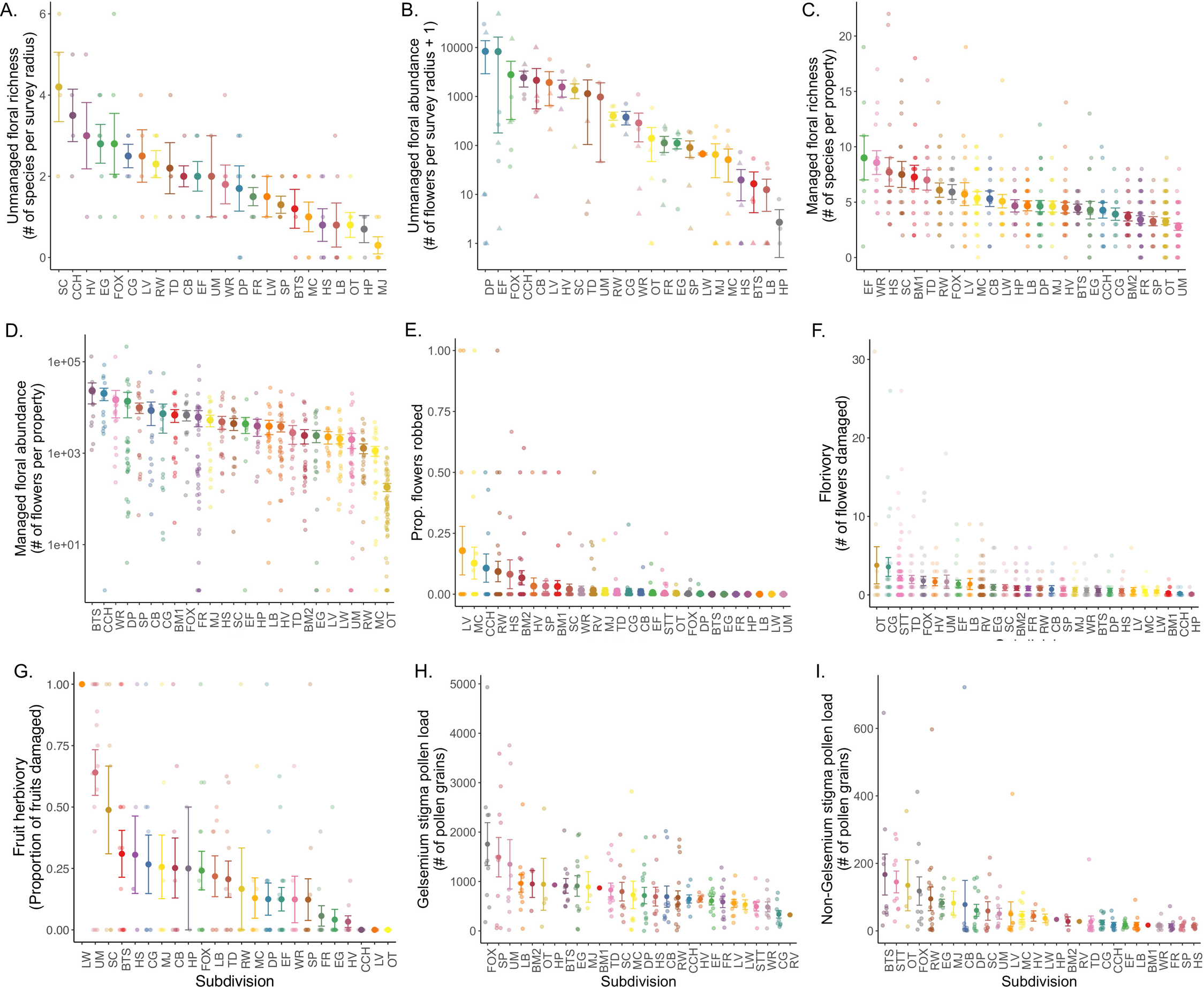

Supplement: S3 Fig — Variation in vegetation attributes (A-D) and interactions (E-I) within and among subdivisions. Large circles and error bars indicate subdivision-level mean value ± 1 s.e.; smaller points indicate individual observations. In each panel, the x-axis is ordered by mean value of the variable of interest. Note log10 transformation of y-axis in B & D. (TIF) [file pone.0312143.s004.tif]
