# Supplementary material for: Socioecological drivers of mutualistic and antagonistic plant-insect interactions and interaction outcomes in suburban landscapes
Source: PLoS One. 2024 Nov 15;19(11):e0312143. doi: 10.1371/journal.pone.0312143 (PMC11567518; doi:10.1371/journal.pone.0312143)
Supplement: Attachment — Submitted filename: Response to reviewers_R1.1.docx [file pone.0312143.s005.docx]

Thanks to the reviewers for their constructive comments. Below, we respond to each comment using **purple text.** Line numbers used in our comments refer to the revised manuscript file *with* track changes visible.

Reviewer #1:

The study addresses an important gap in current research by investigating the influence of socioeconomic and ecological factors on Gelsemium-insect interactions within residential subdivisions. The research methodology is commendable, employing a robust combination of field surveys and statistical analyses to explore complex relationships. Furthermore, the discussion section offers valuable insights into the implications of the findings and suggests promising directions for future research.

Thank you for your positive comments!

One significant area for improvement lies in the clarity of hypothesis testing. While the study presents hypotheses, there is ambiguity in how these hypotheses were tested and addressed in the analysis. Clarifying the hypotheses and their specific relevance to the research objectives would enhance the reader's understanding of the study's purpose and findings.

We have substantially re-worked the section of the manuscript outlining our research objectives and hypotheses, including adding more explanation and justification for them. The revised section of the manuscript can be found in L89-126, as well as additions to the Methods section. (See also our response to comments from Reviewer #2.)

Moreover, the manuscript would benefit from a more detailed explanation of the results. While the results are presented comprehensively, the interpretation sometimes lacks specificity, making it difficult for readers to fully grasp the significance of the observed patterns. Providing deeper insights into the implications of the results and their relevance to the broader research context would strengthen the manuscript's impact.

We have revised the Discussion to improve the interpretation of the results, including an expanded discussion of the implications of the patterns we observed. See the track-change document for full details on these updates.

Additionally, the manuscript would benefit from a more thorough discussion of methodological limitations. While the study design and methods are generally well-described, there is insufficient acknowledgment and exploration of potential limitations. Discussing the limitations inherent in the study design and data collection processes would enhance the credibility of the findings and help contextualize the conclusions within the broader scientific landscape.

In line with your comment here and comments from Reviewer #3, we have revised the manuscript throughout to clarify that the observational approach we used limits our ability to draw inference about causal relationships underlying the patterns we observe. We have also added a section to the Discussion that explicitly discusses some of the limitations of the study (L507-515):

Of course, while observational studies can generate valuable insight into the processes underlying ecological pattern, they cannot determine causality. Particularly in a study such as this, which considered multiple interaction types and environmental parameters, choices about which variables to measure and which to ignore constrain our inference. As we have noted, it is not possible for us to say from this study whether the observed associations represent direct effects of the predictor variable or covariance with another, unmeasured driver. The patterns noted here can serve to guide future investigation, including manipulative experiments that allow for evaluation of causation.

--
Reviewer #2:

a- Species interactions: first of all, this study is not about interactions per se, as there is no data on who visits whom and who eats whom, but it is about the outcome of the (unobserved) interactions, hence about frugivory and pollination: in other words it is about reproductive success. This should be made crystal clear in all parts of the manuscripts, including the title, abstract, aims, hypotheses etc. Results can obviously be interpreted/discussed as a function of changing interactions, but you are not analysing any interaction here! The main flaw of the ms is that it needs to be refocused on what is actually being analysed.

You are certainly correct that we did not measure interactions *per se* in this study. But we think it is incomplete to say that we measured only reproductive success. Instead, we measured both reproductive success (via fruit set and seed production) *and* proxies for interaction frequency (pollen receipt and levels of damage from herbivores, florivores, and nectar robbers). It is common in ecological studies of species interactions to assess proxies of the interaction rather than the interaction itself, and still make inferences about interactions (e.g., for pollination: Tur et al. 2016, *Ecology Letters* [stigma pollen loads]; for herbivory: O’Connor 2009, *Ecology* [plant biomass]). In some cases, measuring this proxy is in fact more informative than measuring the interaction itself. For example, knowing how much of a flower was consumed by florivores is, we would argue, more important to understanding the florivore-plant interaction than knowing how many times a florivore was recorded on the flower. That is, an observation of an interaction occurring is not evidence of its ecological significance – particularly since, for many plant-insect interactions, it can be hard to distinguish between an ecologically trivial interaction (e.g., a pollinator lands on a flower) and a significant one (e.g., a pollinator consumes nectar resources from a flower and in the process transfers pollen to that flower). For these reasons, we think it is reasonable and appropriate to use the term “interactions” to describe the metrics we use here.

That said, we have re-read the manuscript with this comment in mind. To clarify exactly what we measured in this study, we have made the following changes:

L 99-102: As proxies for antagonistic interactions, we assessed the proportion of flowers with evidence of nectar robbery and/or florivory, and the proportion of fruits with herbivore damage; for mutualistic interactions, used pollen receipt as a proxy for pollination.

(previous wording: For antagonistic interactions, we assessed the frequency of nectar robbing and floral and fruit herbivory;…)

Caption for Figure 2 (in part):

Focal plants were censused for evidence of florivory, nectar robbing, and pollination (estimated as pollen deposition), and were assessed for fruit and seed production.

(previous wording: Focal plants were monitored for interactions with florivores, nectar robbers, and pollinators (estimated as pollen deposition), and were assessed for fruit and seed production.)

b- Hypotheses. 98-109 You need to explain why you specifically expect the hypotheses, using literature, theory and/or previous exemplary case studies. Otherwise, these you are just listing just ideas as a brainstorming, which is not a very scientific process. Instead, you may appropriately present the hypotheses with the relevant background of literature, especially considering also what stated in my comment “a”.

We have revised this section to include further explanation and justification for the hypothesized relationships, including citations to relevant literature for each prediction. The revised passage (L102-127) now reads:

We hypothesized that socioeconomic attributes would influence interactions both directly (e.g., by influencing management intensity [26]) and indirectly via their effects on vegetation attributes [27,28], though we did not have *a priori* expectations for the direction of these effects (Fig 1). All the insect species involved in the interactions we assessed are dietary generalists, so we predicted that vegetation attributes such as high managed floral density and richness would increase populations of antagonists and pollinators, and would therefore increase interaction frequency [29,30]. At the same time, greater floral resources can decrease interaction frequency via a dilution effect [31,32]. We predicted that dilution would dominate at local scales (i.e., in the 20m radius around focal plants), leading to negative correlations between unmanaged floral resources and interaction metrics, but that the net effect of landscape-scale floral resource availability would be increased interaction frequency, and therefore a positive association between managed floral resources and interaction metrics (Fig 1). We expected conspecific and heterospecific pollen receipt to be strongly correlated, but predicted that increased non-*Gelsemium* floral density and richness would lead to more pollinator switching and therefore increase heterospecific pollen receipt [33,34]. We expected socioeconomic and vegetation attributes to influence reproductive outcomes for *Gelsemium* only via effects on the frequency of species interactions (rather than direct effects), and that increased interactions with antagonists and heterospecific pollen would reduce reproductive success whereas increased conspecific pollen receipt would increase *Gelsemium* reproduction, given previous evidence for pollen limitation [35]. By integrating social and ecological drivers of plant-insect interactions into a single causal network, we hope to increase mechanistic understanding of the influence of urban development on species interactions.

c- It is not clear in the ms why forest cover should be dependent on property value, age, size. In fact, I think that dependence in the SEM is not justified at all. Forest cover depend on landscape variables mainly and how the urban sprawl was conducted.

Forest cover in this case is a measure of forest cover *within the subdivision*. Therefore, we hypothesized that it would be related to property value, age, and lot size since all these factors are influenced by the subdivision development process. We have explained this with the following addition to the “Socioeconomic and land cover attributes – data sources” section of the Methods (L189-192):

We predicted that forest cover would be related to socioeconomic attributes of subdivisions because choices made during the development process (e.g., house size, lot size) would simultaneously influence property value and remaining forest cover, while age since development would be related to post-development forest regrowth.

See also our discussion of the negative association between subdivision age and forest cover in the Discussion (L449-454):

For example, the negative association between property age and subdivision forest cover likely reflects not an effect of age *per se*, but rather changing regulations and development patterns. In the study region, older subdivisions were built primarily closer to the urban core and tend to be higher density, while newer subdivisions tend to be lower density and include substantial acreage left to woodland.

42 – Here I would directly introduce that cities are an habitat that is different from the non-urban surroundings, see Biella et al 2022 J Apl Ecol. In turn, different areas of a city might be different micro-habitats based on the variation of biotic and abiotic features within a city (CIT).

Thank you for the suggestion. We have added the following sentence where you indicated:

As a result of these changes, urban environments support biological communities that are often distinct from adjacent non-urban counterparts [e.g., 5,6].

47- however, the opposite is much more common actually, with species turnover largely contributing to species interaction change see eg Biella et al 2020 Func Ecol, Carstensen et al 2014 Plos One

We respectfully disagree with this assessment. In our opinion, there is not a clear consensus on the relative importance of species turnover vs. interaction rewiring in determining differences in interaction networks across environmental gradients. For example, in the Carstensen et al. paper you reference, interaction turnover among species shared across sites was of an equal magnitude to species turnover. At any rate, the point of this statement was not to say that interaction turnover is more important than (or even as important as) species turnover in determining network structure, but rather to point out that it has been less thoroughly studied. We stand by this statement, and contend that it is in part an assumption that rewiring without species loss is less significant than species turnover that has led to the relative neglect of the phenomenon.

71-73 but also subdivisions are the same type of habitat… especially from the methods I understood that you are studying forest fragments, hence again the same type of habitat

Subdivisions are the same type of habitat in the same way that cities are the same type of habitat – that is, their similarity can encompass a great deal of variation. The argument we are making here is that within a subdivision, variation in the built environment and associated socioecological attributes is less than in a comparable area of non-master-planned urban environment. This reduction in fine-scale heterogeneity could allow for better inference about the specific socioecological attributes that are driving patterns of association between the urban environment and ecological interactions. This point was made in the lines directly preceding the ones referenced by the reviewer’s comment, and we have not modified it.

91 and elsewhere – it seems a bit out of scope wondering on how vegetation attributes depend on socioeconomic factors: the link between aim 1 and 2 should be better explained. If this paper in on the reproductive success of that specific vine plant, why we are looking at aim1 at all?

We did not intend for this to be read as stating two aims of the paper, but rather to summarize the structure of the model. We see how the previous wording gives the impression of two aims, and so have re-worded the sentence as follows:

We used piecewise structural equation modeling to investigate the links among socioeconomic attributes, vegetation attributes, and the frequency and plant reproductive outcome of antagonistic and mutualistic plant-insect interactions.

To your broader question: with this paper, we are looking at how socioeconomic attributes of suburban areas influence interaction frequency and outcomes. As we detail in the introduction and discussion, there are reasonably well-established links between socioeconomic attributes and managed vegetation in urban residential areas, so we believe assessing these links in our study system is essential for a full understanding of the ways that socioeconomics may influence species interactions.

220-229 pollination and not pollinators

We are uncertain what this comment refers to. The lines referenced detail our methods for assessing nectar robbing. Throughout, we have made efforts to clarify, along the lines of your general comment a, that we assessed stigma pollen load, not pollinator visitation, as our measure of pollination.

289 – predictors are crucial, why in supplementary material? Plase put it in the main text

We have moved Table S2 to the main text, where it is now Table 1.

Table 1 – why forest cover is not in the models of Fruit set and Seed set?

Forest cover was not included because we did not hypothesize a relationship between these variables. Forest cover refers to subdivision-scale extent of forested land, and we saw no reason why this would relate to fruit or seed set. If there was a significant correlation between forest cover and either of these variables, this would have been detected in our test of directed separation, but we found no evidence for these relationships (p>0.3 in both cases).

Results – why property value and plant richness should decrease the “other” pollen? I imagined the opposite.

We were also surprised by the negative association between managed plant richness and heterospecific pollen. While the precise mechanism behind this association requires further investigation, we propose the following explanation in our Discussion:

The contrasting effects of density and richness suggest that while high density of floral resources may increase insect populations and interaction frequency, increased floral species richness may lead to dilution via a sampling effect [47], whereby species-rich communities are more likely to include species highly attractive to potential *Gelsemium* interactors and therefore reduce interaction frequency. (L464-469)

Results – where are the results of aim a?

As we explain in response to your comment referencing L91, we did not intend for the question of the relationship between socioeconomic attributes and vegetation attributes to be considered a separate aim. See our response to that comment with our clarifying edits.

That said, the results pertaining to this question are included in Table 2 and Figure 2 and mentioned in L360-364: “All measured socioeconomic attributes influenced some vegetation attribute. Property age was negatively associated with subdivision forest cover (older subdivisions had less forest cover). Property value was positively associated with managed floral density but not richness, and property size was positively associated with unmanaged floral density but not richness.”

482- and abstract. I have to say that it is not very clear what a subdivision is and why it is relevant as a “special” case, I would ask to explain a bit better the context (above, not here)

Thank you for this observation. We recognize that this term may not be familiar to people outside North America, and have tried to define what a subdivision is, and explain the significance, as follows:

Here we propose and evaluate another approach, taking advantage of the reduced within-development heterogeneity of master-planned residential developments, or subdivisions.

Subdivisions are a common feature of suburban land use in North America, and comprise houses built in the same era, often by a single developer, leading to relative homogeneity in both the built environment and landscaping. Developers’ landscaping choices have long-term legacy effects that can persist for decades [22,23]. Moreover, homeowner associations (HOAs) or similar entities commonly influence landscaping decisions and vegetation management in subdivisions, constraining variation in vegetation structure and composition [24,25]. As such, subdivisions are likely to be more homogeneous in both socioeconomic and environmental conditions when compared with non-master-planned urban neighborhoods. Therefore, subdivisions may provide a ‘natural experiment’ to study effects of specific attributes of urbanization on biotic communities, with each subdivision representing a ‘patch’ with a distinct socioecological profile. Comparing ecological patterns or processes among subdivisions that occupy a common landscape could allow for the identification of causal pathways linking social processes to ecological outcomes.

--

Reviewer #3: I read the Ms “Socioecological drivers of mutualistic and antagonistic plant-insect interactions in suburban landscapes” by Fitch et al. I think this Ms includes a large field and detailed work to explore the correlations between socioeconomic attributes (mean property value, mean property size, subdivision age), vegetation attributes (forest cover and richness and density of managed and unmanaged floral resources), species interactions on a focal native species (Gelsemium; in particular, conspecific and heterospecific pollen deposition, nectar robbing, florivory), and the reproductive traits of this focal plant (fruit set, seeds per fruit).
I have some comments and suggestions that could be considered in the new version.

1) In the introduction there are justifications to relate socioeconomic status and biodiversity. The cited references (32-34) are from the USA and I think to be acknowledge because in some other regions of the world, periurbans with people with low incomes manage biodiversity for food and other uses enhancing biodiversity. I am not questioning the Lines 166-168: “Property value is an indicator of wealth and socioeconomic status, which we predicted would be positively associated with the density and diversity of managed floral resources [32,33]. Property age is related to the types of yard plantings and the amount of time they have been in place – both of which can be related to the availability of managed floral resources [32,34]. Lot size is an indicator of the amount of plantable yard space, and therefore the availability of managed floral resources”; I am just saying this perspective is for a particular region and could not be generalized.

You are certainly correct that the patterns we discuss here are likely to be variable across societies. These predictions are not meant to be applied universally, but refer to our study system. We have clarified that these patterns are not universal by changing the quoted sentence as follows:

“Property value is an indicator of wealth and socioeconomic status, which we predicted would be positively associated with the density and diversity of managed floral resources*, a pattern observed in other North American cities* [34,35].” (italics indicates new addition)

2) In the result section, some “effects” and “influences” are described. For example, Lines 375-378 “The only significant relationship between species interactions and plant reproduction was a strong negative effect of fruit herbivory on seeds per fruit. Other interactions had marginal effects: fruit set and seeds per fruit were both marginally positively influenced by florivory and non-Gelsemium pollen load”. Or in Lines 380-384: “Because property value, managed floral density and richness, and unmanaged floral richness influenced florivory and/or non-Gelsemium pollen load, all these subdivision attributes indirectly (albeit weakly) affected fruit set and seeds per fruit (Table 2). Similarly, seeds per fruit was indirectly influenced by property age via its effect on nectar robbery (Table 2)”. I think the experimental design cannot detect effects or influences between variables. There are correlations between variables (mostly very low negative or positive correlations) that are very interesting to be described but cannot be mentioned as effects or influences.
It is common for observational studies that use SEM analysis to employ the language of “effects” and “influences” to describe relationships among variables (see e.g., Fearon et al. 2022, *Ecology*; Wei et al. 2021, *Nature*). That said, we agree with you that we cannot infer causality from the relationships we document here. We have modified the Results and Discussion throughout, in most cases replacing “influence” and “effect” with “associated with” or “association between”. In a few cases where this led to convoluted and unclear syntax, we have retained the term “influence.”

In addition we have added an additional section to the Discussion that highlights the observational nature of this study, and acknowledges the limitations of such an approach (see our response to the final comment from Reviewer #1).

3) In the Discussion, Lines 389-393 (“We found that socioeconomic attributes influenced both managed and unmanaged floral density but not species richness, while flowering plant richness (both managed and unmanaged) had a greater effect on species interactions. Therefore, direct effects of socioeconomic attributes, and particularly property value, on species interactions were unexpectedly stronger than vegetation-mediated effects”) authors mentioned this statements that are neither reflected by the experimental design nor by their results. Moreover, some lines below mentioned somewhat contradictory sentences (Lines 399-401. Below, we discuss the significant relationships this study uncovered, and why we found relatively little signal of socioecological attributes of subdivisions affecting Gelsemium-insect interactions). I think, these paragraphs could be better aligned using better terms or expanding the explanations?.

We have re-written the quoted section above to reflect that our experimental design does not, strictly speaking, allow for causal inference. That said, we do not believe this contradicts the other statement you quote, later in the paragraph. That is, the relationships we identify in the first section link socioeconomic attributes to floral *density*, while the significant links from floral resources to interactions were only for measures of floral *richness.* Hence, there were only weak floral resource-mediated links between socioeconomic attributes and interaction measures. We have made this distinction clearer in the revised version.

4) The same comment for the introduction when discussing their findings because most previous studies (including one paper with a meta-analysis) were performed in USA and Europe (Lines 402-404. It is well established that socioeconomic status is associated with vegetation composition in residential areas [32,39,40], and the positive effect of property value on managed floral density that we found is consistent with this). I think this trend is for some societies.

Thank you for pointing this out. We have qualified this statement by adding “at least in North America” after “residential areas”.

5) If authors want to include a hypothesis (I think it could not be necessary because this is an exploratory study but very useful), it will be rejected (entirely) or accepted as was presented. The formal proposal to present a hypothesis does not admit a “mixed support”. See Lines 427-431. “We hypothesized that landscape-scale floral resource availability would increase interaction frequency by augmenting insect population size, while co-flowering neighbors would dilute visitation. However, support for this hypothesis was mixed. While managed floral resource density weakly increased stigma pollen loads, there was a stronger opposing negative effect of managed floral species richness on both pollen loads and florivory”.

We think that in this context it is appropriate to talk about “mixed support” for our hypothesis: we predicted that both antagonistic and mutualistic interactions would show similar correlations with managed floral resource availability, but found that the significance and sign of this correlation instead depended on the metric of floral resources used (i.e., density vs. richness) as well as the interaction being assessed. That is, we found some support for our prediction (increased floral resource density was correlated with increased pollen loads, our proxy for interaction with pollinators), but also some opposing responses (floral richness was negatively correlated with pollen load and florivory).

6) Lines 439-452. “Of the socioeconomic attributes we assessed, both property age and property value influenced species interactions…..” I think results do not support this paragraph. Moreover, some lines below (Lines 473-476. “Alternatively, the weak links between socioeconomic attributes and interaction frequency or outcome may reflect scale mismatch, with the socioeconomic variables measured at a larger scale than that at which ecological factors influenced Gelsemium-insect interactions”, text contradicts the paragraph presented above.

Many of the results discussed in this paragraph were marginally significant (i.e., 0.05<p<0.1). Given the sensitivity of some of the constituent submodels in our SEM to precise model formulation, we think that many of these relationships may be biologically relevant and therefore worthy of discussion even if their p-value falls slightly above 0.05 (and even if, in the context of high overall variability, their effects were small). That said, we agree with you that this was not made clear in the paragraph you reference, and so could be confusing to readers. We have substantially revised this paragraph, and now focus on the relationships that did show statistical significance at p<0.05.

Minor comments
Line 152. Correct typo (surveys. census)

Thank you for catching this. Corrected.
