# Supplementary material for: Socioecological drivers of mutualistic and antagonistic plant-insect interactions and interaction outcomes in suburban landscapes
Source: PLoS One. 2024 Nov 15;19(11):e0312143. doi: 10.1371/journal.pone.0312143 (PMC11567518; doi:10.1371/journal.pone.0312143)
Supplement: Attachment — Submitted filename: Response to reviewer_R2.docx [file pone.0312143.s006.docx]

Thanks to Reviewer #3 for their constructive comment. Our response and revision are below.

Reviewer #3: I concur with the updates made in the new edition, except for one aspect.

In science, there are two principal types of hypotheses: (a) direct (inductive), which involves searching for patterns without clear expectations, looking for links, correlations, etc., with the anticipation of discovering distinct patterns post-study, which I believe applies here. The authors stated in the Introduction, "We hypothesized that socioeconomic attributes would influence interactions both directly (e.g., by influencing management intensity [26]) and indirectly through their effects on vegetation attributes." The phrase "would influence" implies no need for trend expectations (i.e., predictions). Conversely, the second type of hypothesis, (b) indirect (deductive), necessitates a solid theoretical framework to underpin the expectations (predictions are mandatory).

In the previous round, I stated (with additional clarifications): 5) If authors wish to include a hypothesis—which may not be necessary in an exploratory study but can be beneficial (thus, an indirect type)—it will either be entirely rejected or accepted as presented. This is because including predictions categorizes it as an indirect type. The formal proposition of a hypothesis (when it is contructed as the indirect type) does not allow for "mixed support."

The indirect type requires full support, while the direct type may accept "mixed support" due to the unpredictable patterns that may emerge from the dataset. This observation aligns precisely with the comment from reviewer #1: “One significant area for improvement lies in the clarity of hypothesis testing. While the study presents hypotheses, there is ambiguity in how these hypotheses were tested and addressed in the analysis. Clarifying the hypotheses and their specific relevance to the research objectives would enhance the reader's understanding of the study's purpose and findings”. Your response for this comment (rev # 1) was: “We have substantially re-worked the section of the manuscript outlining our research objectives and hypotheses, including adding more explanation and justification for them”, and for my comment (rev # 3) was: “We think that in this context it is appropriate to talk about “mixed support” for our hypothesis: we predicted that both antagonistic and mutualistic interactions would show similar correlations with managed floral resource availability, but found that the significance and sign of this correlation instead depended on the metric of floral resources used (i.e., density vs. richness) as well as the interaction being assessed. That is, we found some support for our prediction (increased floral resource density was correlated with increased pollen loads, our proxy for interaction with pollinators), but also some opposing responses (floral richness was negatively correlated with pollen load and florivory)”.

I believe there is a misunderstanding regarding the two types of hypotheses. In your paper, the results and discussion should align with the direct hypothesis introduced earlier, as acknowledged in your responses to the three reviewers. It is unnecessary to "force" the inclusion of predictions if they are not required for the study.

In summary, the rationale of the study must be consistent across all sections of the manuscript, including the Introduction, Materials and Methods, Results, and Discussion.

RESPONSE: To be honest, this distinction in the type of support allowed for direct vs. indirect hypotheses is a new concept to us (with a combined ~100 years of experience among co-authors of conducting hypothesis-testing-oriented research). Thank you for bringing it to our attention.

In this case, we think it is worthwhile to elucidate some more specific predicted relationships, in part to assist the reader in navigating what is otherwise a complicated dataset. Indeed, one response to our initial manuscript draft from both Reviewer #1 and Reviewer #2 was that more explication of specific hypotheses would improve the manuscript.

Therefore, we have opted to retain the specific hypothesized relationships in the manuscript. In recognition of the fact that, therefore, our hypothesis must be either completely accepted or rejected, we have re-written the section of the Discussion where we review our hypothesis as follows (L442-449, change italicized):

Previous research has found neighborhood effects on both antagonistic and mutualistic species interactions to be highly context- and scale-dependent [32,52,53], and our results fit this pattern. *While we found correlations between floral resources and species interactions, they were not consistent across interaction type or floral resource measure, and so did not support our hypothesis that both antagonistic and mutualistic interactions would show similar correlations with managed floral resource availability.* Managed floral resource density weakly increased stigma pollen loads, but there was a stronger opposing negative relationship between managed floral species richness and both pollen loads and florivory.
